# Giemsa-stained pseudo-micronuclei in rat skin treated with vitamin D_3_ analog, pefcalcitol

**DOI:** 10.1186/s41021-017-0077-9

**Published:** 2017-06-01

**Authors:** Akira Takeiri, Kenji Tanaka, Asako Harada, Kaori Matsuzaki, Mariko Yano, Shigeki Motoyama, Chie Katoh, Masayuki Mishima

**Affiliations:** Fuji-Gotemba Research Laboratories, Research Division, Chugai Pharmaceutical Co., Ltd., 1-135 Komakado, Gotemba, Shizuoka 412-8513 Japan

**Keywords:** Pefcalcitol, Vitamin D_3_, Micronucleus, Skin, Rat, Keratohyalin granule, Genotoxicity

## Abstract

**Background:**

Pefcalcitol, an analog of vitamin D_3_ (VD_3_), is an anti-psoriatic drug candidate that is designed to achieve much higher pharmacological effects, such as keratinocyte differentiation, than those of VD_3,_ with fewer side effects. Genotoxicity of the compound was evaluated in a rat skin micronucleus (MN) test.

**Results:**

In the rat skin MN test, pefcalcitol showed positive when specimens were stained with Giemsa, whereas neither an in vitro chromosome aberration test in CHL cells nor an in vivo bone marrow MN test in rats indicated clastogenicity. To elucidate the causes of the discrepancy, the MN specimens were re-stained with acridine orange (AO), a fluorescent dye specific to nucleic acid, and the in vivo clastogenicity of the compound in rat skin was re-evaluated. The MN-like granules that had been stained by Giemsa were not stained by AO, and AO-stained specimens indicated that pefcalcitol did not increase the frequency of micronucleated (MNed) cells. Histopathological evaluation suggested that the MN-like granules in the epidermis were keratohyalin granules contained in keratinocytes, which had highly proliferated after treatment with pefcalcitol.

**Conclusions:**

Pefcalcitol was concluded to be negative in the rat skin MN test. The present study demonstrated that Giemsa staining gave a misleading positive result in the skin MN test, because Giemsa stained keratohyalin granules.

## Background

A micronucleus (MN) test using hematopoietic cells from rodent bone marrow or peripheral blood is an important component of an in vivo genotoxicity assessment [1, 2]. The MN test has been widely used to assess genotoxicity because of its various advantages; it has a clear endpoint and a simple experimental procedure, it does not require expensive laboratory equipment, and it can be applied to various tissues, including liver, gastrointestinal tract, spleen, lung, bladder, buccal mucosa, vagina, and foetal tissues [3]. He and Baker [4] reported that the skin MN test is a useful assay for detecting the genotoxic activity of chemical carcinogens in skin cells, and Nishikawa et al. developed a practical protocol without the ex-vivo cell culture phase, which had previously been part of the skin MN test procedure [5–7]. The skin MN test was able to detect DNA damage caused by photo-reactive chemicals [8], and Itoh et al. reported a significant increase in micronucleated (MNed) skin cells after treatment with quinolones and UV irradiation [9]. Reus et al. made additional optimizations on the method using rats and successfully detected photo-activity of 8-methoxypsoralen [10]. The comet assay can also be used to detect genotoxic and photogenotoxic activity in skin cells [11], and a comparison of the sensitivity and specificity of the skin MN test and the skin comet assay in the same animals demonstrated that the two assays were comparable [12].

Pefcalcitol is a topical application drug candidate with structural modification of 1,25-dihydroxy vitamin D_3_ screened to have superior skin effects as an anti-psoriasis drug [13]. The chemical structure of pefcalcitol is designed to provide quick enzymatic degradation in skin so that concentration of the compound in blood, and consequently the risk of inducing hypercalcemia, can be significantly reduced. As a result, exposure of the skin cells to the compound is 4300-fold higher than the plasma concentration [14]. It would be impossible to achieve this level of skin exposure by an intravenous injection of pefcalcitol in bone marrow, because the systemic exposure would result in a lethal calcium effect. Thus, pefcalcitol represents a typical case when the standard immature erythrocyte MN test is not suitable for evaluating clastogenicity in vivo.

In the present study, we examined the clastogenicity of pefcalcitol by the skin MN test and also by an in vitro chromosomal aberration (CA) test and a bone marrow MN test. A significant increase of MNed cells was observed in the skin MN test with Giemsa staining, but neither clastogenic nor mutagenic activity of the compound was suggested in any other assays. Because it was unlikely that the test compound was clastogenic to rat skin cells only, we re-stained the Giemsa slides with acridine orange (AO), which can react with DNA to cause specific fluorescence. Re-staining with AO revealed that the great majority of the micronuclei (MNi) that had taken up Giemsa stain after treatment with pefcalcitol did not exhibit DNA-specific fluorescence, even though most of the MNi derived from vehicle- or mitomycin C (MMC)-treated rats were stained with AO. The result demonstrated that pefcalcitol induced pseudo-MNi that showed positive with Giemsa staining in rat skin cells to give a misleading score of MNed skin cells.

## Methods

### Chemicals

Pefcalcitol (development code: SMD-502, 2-{[(1*S*,3*R*,5*Z*,7*E*,20*S*)-1,3-dihydroxy-9,10-secopregna-5,7,10(19),16-tetraen-20-yl]oxy}-*N*-(2,2,3,3,3-pentafluoropropyl)acetamide, CAS No. 381212-03-9) was synthesized by Chugai Pharmaceutical Co., Ltd. The purity was >97% when measured with HPLC. Mitomycin C (MMC, CAS No. 50-07-7, Kyowa Hakko Kirin Co., Ltd., Tokyo, Japan) was used as a reference article for a CA test and an MN test. MMC was dissolved in distilled water and diluted with saline for the CA and bone marrow MN tests, or dissolved in a mixture of acetone and olive oil (4:1 by volume) for the skin MN test. Benzo[*a*]pyrene (B[*a*]P, CAS No. 50-32-8, Wako Pure Chemical Industries, Ltd., Osaka, Japan), which was used as a reference article in the CA test, was dissolved in dimethyl sulfoxide (DMSO).

### Animals

Male rats (F344/DuCrlCrlj, Charles River Laboratories Japan, Inc., Yokohama, Japan), 8 weeks old at the time of administration, were used for the skin MN test. Ten-week-old male rats (Crl:CD (SD), Charles River Laboratories Japan, Inc.) were used for the bone marrow MN test. Animal care and experiment procedures were conducted in compliance with the internal regulations for animal use.

### CA test

The CA test was conducted by the routine process. Briefly, CHL/IU cells were treated with the test articles for 24 h without rat liver S9 (Oriental Yeast Co., Ltd., Tokyo, Japan) or for 6 h with or without S9. The 6-h treatments were followed by an 18-h recovery culture. In the test condition of 24-h treatment without S9, the doses were set at 0, 10, 20, and 40 μg/mL. In the 6-h treatment conditions, the doses were set at 0, 12.5, 25, and 50 μg/mL without S9, or 0, 12.2, 36.7, and 110 μg/mL with S9. These doses were set so as to induce at least 50% reduction of cell survival at the highest dose under each test condition. The test article was dissolved in DMSO to prepare 100-fold concentrations of the final test doses. The cells from two culture dishes at each dose were harvested and counted by using Trypan-blue to determine relative cell count (RCC) as an index of cytotoxicity. After a hypotonic treatment with 0.075 mol/L KCl, the cells were fixed with Carnoy’s solution to prepare the slides. Structural and numerical aberrations were microscopically scored on 200 Giemsa-stained metaphase cells at each dose.

### Bone marrow MN test

Pefcalcitol solution (5 mg/mL) was injected via the tail vein in dose volumes of 0.75, 1.5, 3, and 6 mL/kg to achieve dose levels of 3.75, 7.5, 15, and 30 mg/kg, respectively. These doses were selected because a dose of 30 mg/kg was the maximum tolerated dose for rat single dose *i.v.* treatment in a preliminary study (data not shown). MMC was administered intraperitoneally at a dose of 2 mg/kg with a dosing volume of 4 mL/kg. Five male rats per group were used. A solution composed of propylene glycol, ethanol, *N*-methyl-2-pyrrolidinone, and saline (48:6:6:40 by volume) served as a vehicle control and was dosed at 6 mL/kg. Approximately 24 h or 48 h after treatment, bone marrow cells were collected from femurs. Smear specimens of bone marrow from the rats treated with the chemicals or with vehicle were fixed with 99.8% methanol for 5 min and stained with 40 μg/mL AO. MNed erythrocytes among approximately 2000 polychromatic erythrocytes (PCE) were scored at 1000× magnification using fluorescent microscopes (Olympus Corporation, Tokyo, Japan) equipped with a filter set of blue excitation (460–490 nm) and a barrier filter at 515 nm. To evaluate the bone marrow toxicity, reduction in the ratio of PCE to all erythrocytes, which is the total of PCE plus normochromatic erythrocytes (NCE), was measured by counting approximately 1000 erythrocytes.

### Skin MN test

The skin MN test followed the Nishikawa method [5–7], using a slight modification by Reus et al. [10]. An ethanol solution of pefcalcitol prepared at 20 mg/mL was applied onto shaved dorsal skin (3 cm × 4 cm) of rats at a volume of 1 mL/kg, which resulted in a dose of 20 mg/kg. The dose of 20 mg/kg was set because the single dose level was supposed to be sufficient to examine AO stainability of the granules, and the preliminary rat skin MN test (data not shown) showed that pefcalcitol at this dose sufficiently increased the frequency of MN-like granules. The MMC solution at 1 mg/mL was administered in the same manner as pefcalcitol at a volume of 0.2 mL/head to achieve a dose of 0.2 mg/head. Five male rats were used for each dosing group. Skin samples were taken approximately 72 h after treatment. Epidermis cells were harvested through distinct dermo-epidermal separation from the skin samples with cold-digestion in thermolysin (200 μg/mL) followed by isolation with a trypsin solution (phosphate-buffered saline containing 2.5 mg/mL trypsin and 0.4 mg/mL EDTA). The epidermis cells were then swollen with hypotonic 0.075 mol/L KCl solution, fixed with methanol/acetic acid (3:1 by volume), and dropped on to microscope slides after re-suspension in 1% acetic acid methanol solution. The slides were stained with 3% Giemsa in 1/15 mol/L phosphate buffer (pH 6.8) for microscopic analysis, and approximately 2,000 cells per animal were evaluated for the occurrence of MNed cells. The coordinates of each cell with MNi were recorded using a microscope with automated scanning.

### Re-staining of skin MN specimens

After scoring MNed cells, Giemsa dyes were removed from the slide specimens as follows. The slides were immersed in xylene overnight and rinsed with fresh xylene once. Then the slides were immersed in methanol for 1 h, and the immersion was repeated once. After an additional overnight immersion in methanol, the decolorized slides were stained again with 40 μg/mL AO and examined for DNA-specific fluorescence on every MN that had been previously observed under Giemsa staining.

### Histopathological analysis

Skin tissues from the rats treated with vehicle or 20 mg/kg of pefcalcitol were harvested after 72 h of treatment and fixed with 20% neutral buffered formalin. Skin sections prepared from paraffin-embedded blocks were stained with 3% Giemsa solution. Several of the slides were stained with hematoxylin and eosin (HE) solution. The sections from the same paraffin-embedded blocks were stained with 80 μg/mL AO.

## Results

### CA test in CHL cells

**Fig. 1:**
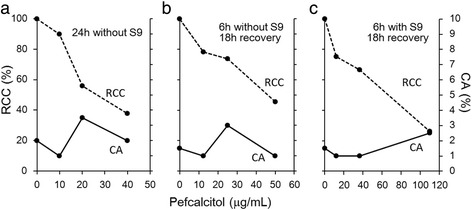
Results of the CA test of pefcalcitol in CHL cells. Percentage of structural chromosomal aberrations (CA%) did not increase with the treatment of pefcalcitol under any treatment conditions: 24-h treatment without S9 derived from rat liver (**a**), 6-h treatment without S9 (**b**), and 6-h treatment with S9 (**c**). Percentages of relative cell counts (RCC%) to vehicle control decreased in a dose-dependent manner, which indicated sufficient exposure of the test chemical

No clastogenic responses were seen in the CA test under any test conditions with or without metabolic activation using rat liver S9 (Fig. 1). No induction of cells with numerical aberrations (polyploidy) was shown (data not shown). Dose-dependent decrease in RCC was shown in each test condition. Minimal RCC was less than 50% at the highest doses in each test condition, which indicated sufficient exposure of the test article. Appropriate positive responses in the cells treated with reference articles were seen. The percentage of cells with structural aberration in the reference articles was 28% (MMC at 0.05 μg/mL), 27% (MMC at 0.1 μg/mL), and 44% (B[*a*]P at 50 μg/mL) under the test conditions of 24-h treatment, 6-h treatment without S9, and 6-h treatment with S9, respectively.

### Bone marrow MN test in rats

**Fig. 2:**
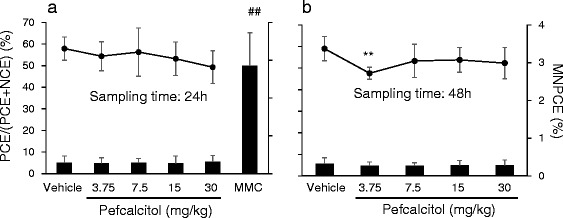
Results of in vivo MN test in rat bone marrow cells obtained 24 h (**a**) or 48 h (**b**) after single intravenous administration of pefcalcitol. The bar represents the incidence of micronucleated polychromatic erythrocytes (MNPCE). The line graph indicates the ratio of polychromatic erythrocytes (PCE) to all erythrocytes, which is the total of PCE plus normochromatic erythrocytes (NCE). MNPCE in 2000 or more PCE of each rat were detected, and the ratio of PCE among 1000 or more total erythrocytes (PCE + NCE) were determined. Each bar or dot represents the average of five or four rats, and error bars of SD are presented. ** *p* < 0.01, significantly different from vehicle control (Student’s *t*-test). ## *p* < 0.01, significantly different from vehicle control (Mann-Whitney U-test). MMC was administered intraperitoneally at a dose of 2 mg/kg as a positive control

No clastogenic responses were seen in the rat bone marrow MN test (Fig. 2). A reduced ratio of PCE to all erythrocytes, which indicates bone marrow toxicity, was not obvious. However, mortality in 2 of 11 rats in the 30-mg/kg group indicated that the exposure to the test chemical was sufficiently high for the MNi to be evaluated. Appropriate MN induction in the positive control group (MMC-treated group) was shown.

### Skin MN test in rats

The results from the skin MN test are shown in Table 1. A significant increase in the frequency of MNed cells was seen from Giemsa staining of the specimens both in the pefcalcitol group (*p* < 0.01) and in the MMC group (*p* < 0.01), either of pefcalcitol- or MMC- induced granules that were interpreted as MNi under Giemsa staining. When the granules interpreted as MNi under Giemsa staining were assessed with AO staining for DNA-specific fluorescence, approximately 60% of the granules induced by pefcalcitol showed no yellow-green fluorescence (Table 1). The mean frequency of MNed cells with fluorescence was 0.44 ± 0.13% in the vehicle control group, which was not different from the previously reported value of 0.18 ± 0.12% [6]. The value in the pefcalcitol group, 0.48 ± 0.10%, was not significantly different from the vehicle-treated group, while in the MMC group, the value of 1.49 ± 0.31% was significantly higher (*p* < 0.01) than the vehicle-treated group. The result suggested that no significant increase in the MNed cells was seen in the pefcalcitol group under AO staining. Therefore, pefcalcitol provided inconsistent results between Giemsa and AO staining.

**Table 1:** Induction of micronuclei in rat skin cells after single topical application of pefcalcitol

Treatment	Micronucleated cells (%)				AO negative/Giemsa positive (%)	
	Giemsa positive		AO positive			
	Individual	Mean ± SD	Individual	Mean ± SD	Individual	Mean ± SD
Vehicle	0.35	0.55 ± 0.14	0.30	0.44 ± 0.13	14	20 ± 6
	0.50		0.40		20	
	0.55		0.40		27	
	0.60		0.45		25	
	0.75		0.65		13	
Pefcalcitol 20 mg/kg	1.15	1.15 ± 0.11**	0.40	0.48 ± 0.10	65	58 ± 11*
	1.15		0.55		52	
	1.00		0.55		45	
	1.30		0.35		73	
	1.15		0.55		52	
Mitomycin C 0.2 mg/body	1.85	1.62 ± 0.29**	1.70	1.49 ± 0.31**	8	8 ± 4
	1.30		1.10		15	
	2.00		1.90		5	
	1.50		1.40		7	
	1.45		1.35		7	

*p < 0.05, **p < 0.01, significantly increased from vehicle control value (Mann-Whitney U-test)

### Histopathological analysis

**Fig. 3:**
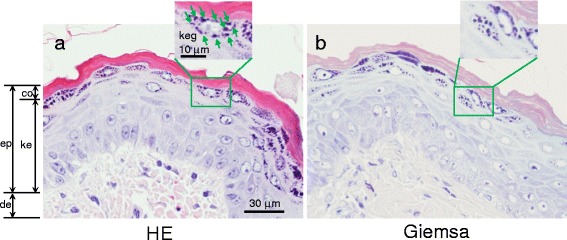
Representative images of the pefcalcitol-treated rat skin stained with HE (**a**) or with Giemsa (**b**), showing that both HE and Giemsa stained keratohyalin granules in the keratinocyte layer. Epidermis (ep), dermis (de), cornified layer (co), and a keratinocyte layer (ke) consisting of granular, basal, and squamous layers are indicated. *Green* arrows point to keratohyalin granules (keg)

**Fig. 4:**
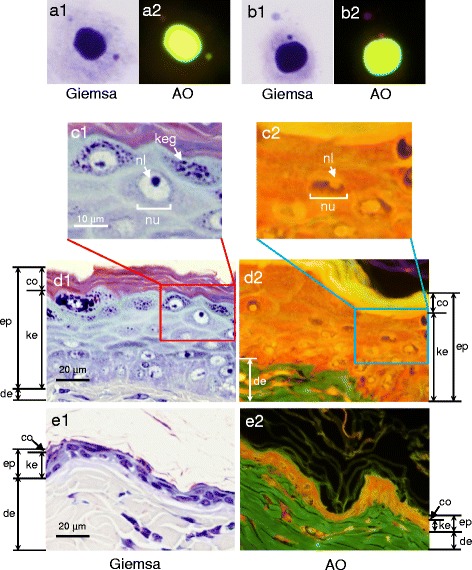
Representative images of a true MNi that had been induced by pefcalcitol treatment in rat skin, stained with Giemsa (A1) and with AO (A2), contrasted with a pseudo-MNi that became stained with Giemsa (B1), but not with AO (B2). Histopathological changes in rat skin tissues 72 h after dermal application of pefcalcitol (C and D) or vehicle (E). Sections were prepared by the routine formalin-paraffin method and stained with Giemsa (C1, D1 and E1) or AO (C2, D2 and E2). Epidermis (ep); dermis (de); cornified layer (co); a keratinocyte layer (ke) consisting of granular, basal, and squamous layers; nucleus (nu); nucleolus (nl); and keratohyalin granules (keg) are indicated. Keratohyalin granules in the keratinocyte layer were stained obviously with Giemsa but not with AO. The staining density of nuclei on the histopathology slides (C1 and C2) was less than that on the slides for MN observation (A1, A2, B1, and B2) because the sliced section was generally thinner than the width of a nucleus

A slide stained with HE indicated that keratohyalin granules in the outer layer of keratinocytes were obvious in pefcalcitol-treated skin of rats (Fig. 3a). A slide stained with Giemsa showed dark blue cytoplasmic granules in the keratinocytes (Fig. 3b). Morphologies and loci of the granules were very similar to those of the keratohyalin granules stained with HE (Fig. 3a), which strongly suggested that the granules stained with Giemsa were keratohyalin granules. Although a few granules were seen in the vehicle-treated rats (Fig. 4, E1), the granules were more obviously induced after treatment with pefcalcitol (Fig. 4, C1 and D1). Pefcalcitol caused hypertrophic change in the epidermis layers, including the cells with the granules (Fig. 4, D1). Staining of the keratohyalin granules by AO was obscure (Fig. 4, C2 and D2). Staining density of nuclei on the histopathology slides (Fig. 4, C1 and C2) was less than that on the slides for MN observation (Fig. 4, A1, A2, B1, and B2) because the sliced section was generally thinner than the width of a nucleus.

## Discussion

Inconsistent results were provided by Giemsa and AO staining in the skin MN test on pefcalcitol. Pefcalcitol significantly increased MNi with Giemsa staining, but when the MNi seen under Giemsa staining were re-stained with AO, more than half of the MNi did not emit DNA-specific yellow-green fluorescence (Fig. 4, B1 and B2). As calculated from MN scores with Giemsa and AO (Table 1), AO-negative percentages of Giemsa MN were 20% (0.11/0.55) in the vehicle control group, 58% (0.67/1.15) in the pefcalcitol group, and 8% (0.13/1.62) in the MMC group. Pefcalcitol significantly induced AO-negative MNi under the test condition, whereas MMC-induced MNi were almost all AO-positive.

Giemsa dye, which is composed of eosin, azure B, and methylene blue, stains various cellular components with gradation from red to dark blue. On the other hand, AO molecules intercalated into double strand DNA emit specific yellow-green fluorescence under 492 nm excitation. Therefore, true MNi composed of chromosomal proteins and DNA are stained violet with Giemsa and yellow-green with AO. Because pefcalcitol increased AO-negative MNi only, the significant increase in MNi with Giemsa staining is not indicative of chromosomal damage.

The maximum plasma concentration of pefcalcitol was 1.7 ng/mL after 20 mg/kg dermal administration to rats (data not shown). Tissue distribution measurements in mice showed that skin concentration was approximately 4,300-fold higher than plasma concentration after 0.5 mg/kg dermal application of pefcalcitol [14]. Using a 4,300-fold factor from plasma to skin, target tissue exposure of pefcalcitol in the present skin MN study was estimated as 7.3 μg/mL (per g of tissue). The average plasma concentration up to 24 h (AUC_0–24_/24 h) for pefcalcitol was 0.23 μg/mL in rats after a single *i.v.* injection at 15 mg/kg [14]; therefore, the estimated skin exposure level in the present study was 32 times higher than the *i.v.*-treated bone marrow MN test. However, the results between the skin MN test (positive) and *i.v.*-treated bone marrow MN test (negative) cannot be attributed to the exposure difference, because a negative result was returned by the CA test, which had much higher exposure levels than these MN tests. The highest concentrations in the CA assay were 110 (6-h treatment with S9), 50 (6-h treatment without S9), and 40 μg/mL (24-h treatment without S9) (Fig. 1), which are approximately 15, 7, and 5 times higher than the skin exposure in the present skin MN test. Moreover, pefcalcitol elicited no genotoxic responses in the Ames test, the *gpt* delta mouse mutation assay with skin and liver tissues after dermal application, or the in vitro mutation assay with GDL1 cells [14], which further support the lack of genotoxicity of pefcalcitol. Taken together, the observation of increased skin MNi with Giemsa staining was concluded to be a false positive.

Morphological changes in the epidermis after treatment with pefcalcitol are indicated in Fig. 4, C1 and D1. Pefcalcitol strongly accelerates the proliferation and differentiation of skin cells. The dark blue granules seen in the cytoplasm of cells located in the outer layer of the keratinocytes were considered to be keratohyalin granules because their hematoxylin-stainability, location, and morphology match previously reported examples [15]. Because some of the granules look similar in size and color to the MNi in this study (Fig. 4, A1 and C1), it is reasonable to consider that the induction of keratohyalin granules might affect the skin MN scoring in epidermis cells under the test conditions in this study.

Because Giemsa and AO staining have their own advantages, both are widely used in MN tests. For dermal application studies, Nishikawa et al. [5–7] used AO staining while Reus et al. [10] used Giemsa staining. We performed a skin MN test with Giemsa staining and observed a false positive result after the dermal treatment with pefcalcitol. The present study demonstrates that pefcalcitol stimulated the proliferation and differentiation of skin cells and induced pseudo-MNi that responded to Giemsa staining, which gave a misleading result in the genotoxicity assessment in a skin MN test.

## Conclusions

The Giemsa-stained MN-like granules that were induced by pefcalcitol treatment in rat skin were not stained by AO, and pefcalcitol was concluded to be negative in the rat skin MN test. The MN-like granules were suggested to be keratohyalin granules in the keratinocytes. The present study demonstrates that Giemsa staining may give misleading positive results in skin MN tests because of interference with MN scoring by keratohyalin granules.

## Abbreviations

AO: Acridine orange
AUC: Area under the curve
B[*a*]P: Benzo[*a*]pyrene
CA: Chromosomal aberration
DMSO: Dimethyl sulfoxide
EDTA: Ethylenediaminetetraacetic acid
HE: Hematoxylin eosin
MMC: Mitomycin C
MN: Micronucleus
MNed: Micronucleated
MNi: Micronuclei
MNPCE: Micronucleated polychromatic erythrocytes
NCE: Normochromatic erythrocytes
PCE: Polychromatic erythrocytes
RCC: Relative cell count
VD_3_: Vitamin D_3_

## References

[1] ICH steering committee (2011). ICH harmonised tripartite guideline, guidance on genotoxicity testing and data interpretation for pharmaceuticals intended for human use S2 (R1).

[2] Hayashi M (2016). The micronucleus test -most widely used *in vivo* genotoxicity test-. Genes Environ.

[3] Morita T, MacGregor JT, Hayashi M (2011). Micronucleus assays in rodent tissues other than bone marrow. Mutagenesis.

[4] He SI, Baker RS (1989). Initiating carcinogen, triethylenemelamine, induces micronuclei in skin target cells. Environ Mol Mutagen.

[5] Nishikawa T, Haresaku M, Adachi K, Masuda M, Hayashi M (1999). Study of a rat skin *in vivo* micronucleus test: data generated by mitomycin C and methyl methanesulfonate. Mutat Res.

[6] Nishikawa T, Haresaku M, Fukushima A, Nakamura T, Adachi K, Masuda M (2002). Further evaluation of an *in vivo* micronucleus test on rat and mouse skin: results with five skin carcinogens. Mutat Res.

[7] Nishikawa T, Nakamura T, Fukushima A, Takagi Y (2005). Further evaluation of the skin micronucleus test: results obtained using 10 polycyclic aromatic hydrocarbons. Mutat Res.

[8] Hara T, Nishikawa T, Sui H, Kawakami K, Matsumoto H, Tanaka N (2007). *In vivo* photochemical skin micronucleus test using a sunlight simulator: detection of 8-methoxypsoralen and benzo[a]pyrene in hairless mice. Mutat Res.

[9] Itoh S, Katoh M, Furuhama K (2002). *In vivo* photochemical micronucleus induction due to certain quinolone antimicrobial agents in the skin of hairless mice. Mutat Res.

[10] Reus AA, Usta M, van Meeuwen RN, Maas WJ, Robinson SA, Kenny JD (2010). Development and characterization of an *in vivo* skin photomicronucleus assay in rats. Mutagenesis.

[11] Fullove TP, Yu H (2013). DNA damage and repair of human skin keratinocytes concurrently exposed to pyrene derivatives and UVA light. Toxicol Res (Camb).

[12] Toyoizumi T, Ohta R, Kawakami K, Nakagawa Y, Tazura Y, Kuwagata M (2012). Usefulness of combined *in vivo* skin comet assay and *in vivo* skin micronucleus test. Mutat Res.

[13] Shimizu K, Kawase A, Haneishi T, Kato Y, Kinoshita K, Ohmori M (2006). Design and evaluation of new antipsoriatic antedrug candidates having 16-en-22-oxa-vitamin D_3_ structures. Bioorg Med Chem Lett.

[14] Takeiri A, Tanaka K, Shioda A, Harada A, Yano M, Kawase A (2012). Lack of mutagenicity of SMD-502, a new vitamin D_3_ analog for topical application, in skin and liver of *gpt* delta transgenic mice and in GDL1 cells. Genes Environ.

[15] Fukuyama K, Kakimi S, Epstein W (1980). Detection a fibrous component in keratohyalin granules of newborn rat epidermis. J Invest Dermatol.

